# Serum IL-33 Is a Novel Diagnostic and Prognostic Biomarker in Acute Ischemic Stroke

**DOI:** 10.14336/AD.2016.0207

**Published:** 2016-10-01

**Authors:** Qian Li, Yuanshao Lin, Wensi Huang, Yulei Zhou, Xiaoli Chen, Brian Wang, Wanli Zhang, Zhengyi Cai, Jie Xue, Wenhui Zhang, Tieer Yu, Hong Wang, Jincai He, Kunlin Jin, Bei Shao

**Affiliations:** ^1^Zhejiang Provincial Key Laboratory of Aging and Neurological Disorder Research,; ^2^Department of Clinical Laboratory Medicine, First Affiliated Hospital, Wenzhou Medical University, Wenzhou 35000, China; ^3^Institute for Healthy Aging, Center for Neuroscience Discovery, University of North Texas Health Science Center, Fort Worth, Texas 76107, USA

**Keywords:** interleukin-33, acute ischemic stroke, outcome, biomarker, diagnosis

## Abstract

Interleukin-33 (IL-33), a newly recognized IL-1 family member, is expressed in various tissues and cells, and involved in pathogenesis of many human diseases. For example, IL-33 plays a protective role in cardiovascular diseases. However, the role of IL-33 in acute ischemic stroke (AIS) remains unclear. This study aims to investigate whether IL-33 level in AIS patient serum can be used as a potential diagnostic and prognostic marker. The study included two hundred and six patients with first-ever ischemic stroke, who were admitted within 72 hours after stroke onset. The serum level of IL-33 was measured with ELISA and the severity of AIS patients on admission was evaluated based on the National Institutes of Health Stroke Scale (NIHSS) score. The functional outcome at 3 months was determined using the Barthel index (BI). We found that serum IL-33 was significantly higher (*P* < 0.001) in patients with AIS [57.68 ng/L (IQR, 44.95-76.73)] compared with healthy controls [47.48 ng/L (IQR, 38.67-53.78)]. IL-33 was an independent diagnostic biomarker for AIS with an OR of 1.051 (95%Cl, 1.018-1.085; P=0.002). Serum IL-33 was higher (*P* < 0.05) in the stroke patients with small cerebral infarction volume compared to AIS patients with large cerebral infarction. In addition, serum IL-33 was also significantly higher (*P* = 0.001) in the patients with mild stroke, compared to the patients with severe stroke. Furthermore, serum IL-33 level in AIS patients with a worse outcome was higher (*P* < 0.001) compared to AIS patients with a better outcome. IL-33 was also an independent predictor for the functional outcome with an adjusted OR of 0.932 (95% CI, 0.882-0.986). Our results suggest that the lower level of serum IL-33 is associated with large infarction volume and greater stroke severity in AIS patients. Thus, IL-33 can be used as a novel and independent diagnostic and predicting prognostic marker in AIS.

Stroke is the second killer in the world in those greater than 60 years of age. In China, with a population of 1.4 billion, the occurrence of new stroke cases is 2.5 million per year. The stroke mortality rate has reached nearly 1.6 million a year, resulting in stroke exceeding heart disease to become the leading cause of death and adult disability in China.

Stroke causes a significant burden on human health [1-3]. Ischemic stroke (IS) accounts for nearly 80-85% of all stroke cases and is caused by the interruption of cerebral blood flow because of a blood clot [2, 4]. Early control of risk factors and evaluation of the severity of the disease and prognosis are important for effective care to improve functional outcome [1].

There is large body of evidence supporting the crucial role of inflammation in the pathophysiologic processes of acute ischemic stroke (AIS) [5, 6]. When AIS occurs, inflammatory responses occur both in the peripheral and central nervous systems (CNS). After the onset of ischemic stroke, the inflammatory cascade results in the damage of the blood-brain barrier (BBB), which then allows activated peripheral immune cells such as neutrophils and T-cells to penetrate and accumulate in the ischemic brain region [7]. Many recent studies have suggested that inflammation may increase tissue injury, but may also be beneficial to the reparative process [8, 9]. After cerebral ischemia, inflammatory responses both in and out of the brain promote brain inflammation by producing inflammatory mediators such as cytokines that promote scavenging necrotic debris after AIS [8, 10]. Growing evidence shows that decreasing pro-inflammatory cytokines and increasing anti-inflammatory cytokines are correlated with a smaller infarct size and better clinical outcome [11], suggesting that the balance between inflammatory and anti-inflammatory cytokines determines the susceptibility and prognosis in AIS. Thus, biomarkers of pro-inflammatory or anti-inflammatory cytokines can be an early diagnostic and prognostic indicator of ischemic stroke. Yet, an available biomarker has not been identified.

Interleukin-33 (IL-33) is a member of the IL-1 cytokine family discovered in 2003. IL-33 has dual functions, one being a transcription factor combined with chromosomes and another being a traditional cytokine considered to be an ‘alarmin’ to danger signals [12, 13]. IL-33 plays an important role in inflammation. IL-33 interacts with its heteromeric receptor composed of ST2 and the IL-1 receptor accessory protein (IL-1RAcp) [14, 15] to enhance Th2-type immune responses [16-19]. The IL-33/ST2 pathway is involved in various human diseases including asthma, inflammatory bowel disease, diabetes, autoimmune disease and cerebral inflammation [20-22].

Yet, IL-33 also has various protective functions in cardiovascular and Th1-mediated inflammatory diseases, and it does so by tilting the Th1/Th2 balance in favor of Th2 cells [23, 24]. Savas Guzel *et al* [25] found that IL-33 plays a protective role during the early stage of non-ST elevated myocardial infarction. In mice with diabetes mellitus, the lower expression of IL-33 may exaggerate ischemia/reperfusion-induced myocardial injury [26]. Thus, IL-33 has an effective influence in regulating the inflammatory response.

Several studies have found that IL-33 is widely expressed in the brain and spinal cord, suggesting that IL-33 may have specific functions in the central nervous system (CNS) [13, 27]. Recently, studies also reported that IL-33 has been implicated in certain CNS diseases such as Alzheimer disease (AD) and experimental autoimmune encephalo-myelitis (EAE). One recent study showed that the IL-33 gene single nucleotide polymorphism might have a relationship with ischemic stroke in the population residing in the northern part of China [28]. Thus, IL-33 may have a role in the progression of AIS.

The role of IL-33 in cerebral ischemic infarction is still controversial. In animals, Luo Y *et al* [24] demonstrated that IL-33 in the brain could improve ischemic brain injury via tilting the Th1/Th2 balance in favor of Th2 cells and suppress Th17 response in mice, suggesting that IL-33 has a protective effect for AIS. In humans, Liu J *et al* [20] showed that the change in serum IL-33 level was positively correlated with the infarct volume in AIS. It was suggested that the circulating IL-33 is associated with AIS and may be involved in the regulation of inflammatory reaction in cerebral infarction. These findings indicate that IL-33 may be a marker of diagnosis or prognosis in stroke. Whether it may increase tissue injury or promote healing in AIS is not clear.

To date, there is no available study on the relationship between IL-33 and functional outcome of AIS patients. Therefore, this study aims to investigate whether the level of IL-33 in AIS patient serum can be used as a potential diagnostic and prognostic marker.

## METHODS AND MATERIALS

### Patients and Clinical Varies

206 first-ever acute ischemic stroke patients (75 females and 131 males; average age of 61.2 ± 10.4 years) were recruited from January 1, 2014 to May 31, 2015 at The First Affiliated Hospital of Wenzhou Medical University, China. All patients were diagnosed as having an AIS according to the World Health Organization criteria [29] and have had symptoms within 72 h. 81 age and sex-matched healthy individuals were selected from the Health Physical Examination Center of The First Affiliated Hospital of Wenzhou Medical University and these subjects constituted the healthy control group (46 males and 35 females; average age of 58.8 ± 14.6 years). Patients with the following conditions were excluded: heart failure, history of trauma, surgery or trauma within the last 2 months, renal dysfunction, liver insufficiency, serious infections, as well as Th2-related diseases such as asthma, atopic dermatitis, and anaphylaxis.

The study was approved by the ethics committee of the First Affiliated Hospital of Wenzhou Medical University. All participants or their relatives were informed of the study and signed the consent forms before inclusion in the study.

**Table 1:** Baseline characteristics of patients with favorable or unfavorable outcomes

Characteristics	Patients	Favorable Outcome	Unfavorable Outcome	*p^a^*
N	206	146	44	
Age (years), Median (IQR)	63 (52-69)	62 (52-67)	64 (55-71)	0.036
Sex (no.):				< 0.001
Male	131	104	16	
Female	75	42	28	
Systolic blood pressure	162 (147-178)	160 (146-177)	170 (149-186)	0.096
Diastolic blood pressure	84 (76-94)	84 (76-94)	86 (75-96)	NS
Median NIHSS score (IQR)	4 (2-7)	3 (2-5)	9 (7-11)	< 0.001
Infarction volume (mL IQR n=175)	1.27 (0.53-4.80)	1.1 (0.40-3.41)	5.10 (1.66-7.56)	< 0.001
Risk factors (no)				
Hypertension	158	111	37	0.066
Diabetes mellitus	46	31	13	0.035
Hypercholesterolemia	55	39	12	NS
Atrial fibrillation	22	19	1	0.035
Smoking	83	65	10	0.011
Alcohol abuse	56	48	2	< 0.001
Laboratory findings (median, IQR)				
WBC (10^9^/L)	6.58 (5.50-8.03)	6.60 (5.46-7.99)	6.37 (5.81-7.77)	NS
Lithic acid (μmol/L)	285.00 (226.00-366.50)	291.00 (234.00-369.00)	257.00 (208.25-341.75)	NS
Vit D (nmol/L)	57.31 (38.73-76.23)	58.53 (39.67-78.61)	51.73 (36.75-74.82)	NS
TC (mmol/L)*	4.94 ± 1.05	4.93 ± 1.04	4.99 ± 1.03	NS
TG (mmol/L)	1.57 (1.15-2.28)	1.61 (1.21-2.42)	1.56 (1.16-2.04)	NS
HDL (mmol/L)	1.08 (0.94-1.29)	1.06 (0.93-1.27)	1.12 (1.00-1.32)	NS
LDL (mmol/L)*	2.92 ± 0.86	2.90 ± 0.85	3.05 ± 0.88	NS
Glucose (mmol/L)	4.90 (4.40-6.15)	4.90 (4.40-5.83)	5.30 (4.65-8.35)	0.044
IgG (mg/mL)	12.00 (10.70-13.80)	11.75 (10.60-13.80)	13.15 (10.88-14.05)	NS
IgA (mg/mL)	2.27 (1.71-3.13)	2.20 (1.63-3.13)	2.61 (2.22-3.26)	0.019
IgM (mg/mL)	0.99 (0.78-1.38)	0.97 (0.78-1.43)	1.16 (0.76-1.54)	NS
Hs-CRP (mg/L)	1.82 (0.70-3.99)	1.79 (0.74-3.69)	4.13 (2.20-8.06)	< 0.001
HbA1c (%)	5.80 (5.50-7.10)	5.80 (5.50-6.48)	6.35 (5.80-8.13)	0.013
IL-33 (ng/L)	57.68 (44.95-76.73)	62.53 (47.19-81.34)	49.83 (36.20-61.22)	< 0.001

Data are presented as the median (IQR) or

* mean (standard deviation). IQR, Interquartile range; NIHSS, National Institutes of Health Stroke Scale; WBC, Leukocyte; Vit D, Vitamin D; TC, Total cholesterol; TG, Triglycerides; HDL, High-density lipoproteins; LDL, Low-density lipoproteins; Ig A, Immunoglobulin A; Ig G, Immunoglobulin G; Ig M, Immunoglobulin M; Hs-CRP, High-sensitivity C-reactive protein; HbA1c, Glycated hemoglobin; IL-33, Interleukin-33; NS, Not significant. *p^a^* value was assessed using the Mann-Whitney U test or the Chi-Square test.

### Data Collection

The following basic clinical information was collected: age, sex, medical history of risk factors (hypertension, hyperlipidemia, diabetes mellitus, atrial fibrillation, smoking habit, and alcohol abuse) and several biochemical indices. All participants were examined with Magnetic Resonance Imaging (MRI) or Computed Tomography (CT) scans. The infarct area was calculated using the formula 0.5 x a x b x c (a: the maximal longitudinal diameter; b: the maximal transverse diameter perpendicular to a; c: the number of 10-mm slices containing the infarct) [30]. A small infarct volume was defined asless than 5 cm^3^. The severity of AIS patients was determined on admission using the National Institutes of Health Stroke Scale (NIHSS) score [31]. Functional outcome was measured at 3 months after admission using the Barthel index (BI) [32] blinded to IL-33 levels. An unfavorable outcome was defined as a score below 85 on the Barthel Index (BI).

### Blood Collection and Laboratory Test

Blood samples of all participants were collected at 6:00 a.m. after the admission. Samples were immediately centrifuged and aliquots were stored at -80°C for further analysis. Serum IL-33 levels were measured by means of enzyme-linked immunosorbent assays (ELISA) [Boyun Biotech Co, Shanghai, China]. The range of the standard curve for IL-33 was 0.5-200 ng/L. Other parameters such as white blood cell count (WBC), Vitamin D (Vit D), Total cholesterol (TC), Triglycerides (TG), High-density lipoproteins (HDL), Low-density lipoproteins (LDL), Immunoglobulin A (IgA), Immunoglobulin G (IgG), Immunoglobulin M (IgM), C-reactive protein (Hs-CRP), and Glycated hemoglobin (HbA1c) were also tested using ELISA.

### Statistical Analysis

Statistical analysis was performed with SPSS 19.0 (SPSS Inc., Chicago, IL, USA). The results were expressed as percentages for categorical variables and as means in normally distributed variables or medians in nonnormally distributed variables. Continuous variables were compared using the Mann-Whitney test between groups and the Chi-Square test was used to compare proportions. The relation between serum IL-33 levels and functional outcome in AIS was computed by multivariate logistic regression analysis, after adjusting for the confounders in the univariate analyses (age, sex, NIHSS score, infarction volume, hypertension, hyperlipidemia, diabetes mellitus, atrial fibrillation, smoking habit, alcohol abuse, and several biochemical indices). Results are expressed as adjusted OR (odds ratio) with the corresponding 95% CI (Confidence Interval). The accuracy of blood biomarkers to diagnose and serve as a prognosis for AIS was determined by the receiver operating characteristic (ROC) curves. The area under the curve (AUC) was calculated as a criterion for the accuracy of the test. Statistical significance was set at *P* < 0.05.

**Figure 1.**
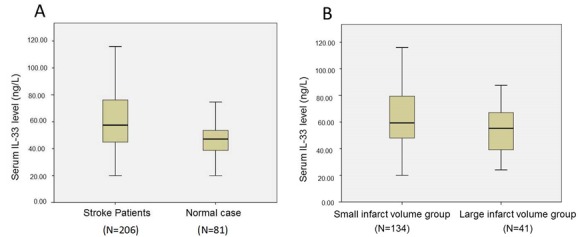
**Serum levels of IL-33 in different groups.** (**A**) Serum levels of IL-33 in IAS Patients and healthy controls. (**B**) Serum levels of IL-33 in small and large infarct volume groups. Statistical comparisons were made using the Mann-Whitney test. **A:** *P* < 0.001; **B:** *P* < 0.05.

## RESULTS

### Baseline Characteristics of Study Samples

In our study, 206 patients with AIS were recruited and 190 patients had the 3 months follow-up (10 patients or their relatives refused to offer information and 6 patients failed to be contracted). The median NIHSS score on admission was 4 points (IQR, 2-7). An unfavorable outcome was found in 44 patients (23.16%) with a median BI score of 92 (IQR, 90-100) (Table 1).

### IL-33 and clinical variables

We found the median serum IL-33 concentration was significantly higher (*P* < 0.001) in AIS patients compared to healthy controls (Fig. 1A). There was a weak but significant negative correlation between IL-33 and Hs-CRP (r = -0.178, *P*=0.011). There was no effect of age, sex, risk factors of stroke, WBC, glucose, lithic acid, TG, TC, HDL, LDL, IgG, IgA, IgM, and HbA1c on IL-33 on AIS patients (*P*>0.05 for all categories).

In univariate logistic regression analysis, IL-33 was consistent with a predictor of AIS with an unadjusted OR of 1.048 (95% CI, 1.030-1.067; *P* = 0.002). Serum IL-33 level was an independent predictor for AIS with an OR of 1.051 (95% CI, 1.018-1.085; *P* < 0.001) after adjusting for all other significant factors (systolic blood pressure, lithic acid, diastolic blood pressure, WBC, TC, HDL, glucose, HbA1c, and Hs-CRP). In addition, glucose, lithic acid, HDL, HbA1c, and Hs-CRP were independent predictors for AIS. Based on the ROC curve, the optimal cutoff value of serum IL-33 levels as an index to diagnose AIS was projected to be 47.92 ng/L. It produced a sensitivity of 0.699 and a specificity of 0.444 with an area under the curve of 0.706 (95% CI, 0.645-0.767; *P* < 0.001). Therefore, IL-33 showed a significantly greater discriminatory ability compared with glucose, Lithic acid, HDL, HbA1c, Hs-CRP (Table 2).

**Table 2:** Accuracy of serum biomarkers in stroke

Prediction	AUC	95% CI	*P*
Glucose	0.332	0.269-0.394	< 0.001
Lithic acid	0.352	0.282-0.423	< 0.001
HDL	0.247	0.187-0.307	< 0.001
HbA1c	0.656	0.588-0.724	< 0.001
Hs-CRP	0.640	0.563-0.717	= 0.001
IL-33	0.706	0.645-0.767	< 0.001

AUC, Area under the curve; CI, Confidence interval; HDL, High-density lipoproteins; HbA1c, Glycated HDL, High-density lipoproteins; HbA1c, Glycated hemoglobin; Hs-CRP, High-sensitivity C-reactive protein; IL-33, Interleukin-33.

### IL-33 and the cerebral infarction volume

175 patients’ MRI or CT scans were available. We found that the level of serum IL-33 was significantly higher (*P* < 0.05) in the small cerebral infarction volume patients group, compared with the large cerebral infarction volume patients group (Fig. 1b). Moreover, serum IL-33 in the small and large infarct volume groups were significantly higher than the control group (Table 3).

### IL-33 and the severity of AIS

The patients were divided into two groups according to the National Institutes of Health Stroke Scale (NIHSS) score: 153 patients with mild stroke (NIHSS score < 6) and 53 patients with moderate to severe stroke (NIHSS score ≥ 6). We found that the level of serum IL-33 was significantly higher (*P* = 0.001) in the mild stroke patients, compared to the severe stroke patients. Serum IL-33 level in the mild stroke groups was significantly higher than the control group (*P* < 0.001). However, there was no significant difference in serum IL-33 level between the moderate to severe stroke group and the control group (*P*>0.05) (Table 4).

### IL-33 and 3-month functional outcome

An unfavorable outcome was found in 44 patients (23.16%) with a BI score below 85. Characteristics of patients with favorable and unfavorable outcomes are provided in Table 1. We found that serum IL-33 levels were significantly higher in patients with a favorable outcome [62.53 ng/L (IQR, 47.19-81.34)] compared with patients with an unfavorable outcome [49.83 ng/L (IQR, 36.20-61.22)] (Table 1).

**Table 3:** Serum interleukin-33 levels in different infarction volume and control groups

Group	N	Median, ng/L (IQR)
Small infarct volume	134	59.36 (47.92-79.51)^a^, ^b^
Large infarct volume	41	55.72 (39.24-67.23)^c^
Control	81	47.48 (38.67-53.78)

Data are presented as median (IQR)

a *p*< 0.001, compared with the control group.

b *p* = 0.036, compared with the large infarct volume group.

c *p* = 0.011, compared with the control group.

Compared with NIHSS and other risk factors as presented in Table 5, we found that serum IL-33 level was associated with a favorable outcome using univariate logistic regression analysis with an unadjusted OR of 0.952 (95% CI, 0.931-0.973). After adjusting for all other significant outcome predictors (age, sex, systolic blood pressure, NIHSS score, hypertension, atrial fibrillation, diabetes mellitus, smoking, alcohol abuse, lithic acid, Vit D, HbA1c, IgA, glucose, Hs-CRP), IL-33 was still an independent predictor for outcome with an adjusted OR of 0.932 (95% CI, 0.882-0.986). In addition, sex, NIHSS score, and Hs-CRP were independent predictors for stroke outcome (Table 5).

**Table 4:** Serum interleukin-33 levels in mild and severe stroke patients and healthy controls

Group	N	Median, ng/L (IQR)
Mild stroke group	153	60.32 (47.43-79.65)^a^,^b^
Severe stroke group	53	52.77 (37.45-64.16)^c^
Control	81	47.48 (38.67-53.78)

Data are presented as median (IQR)

a *p* < 0.001, compared with the control group.

b *p* = 0.001, compared with the severe stroke group.

c *p* = 0.076, compared with the control group.

**Table 5:** Univariate and multivariate logistic regression analysis for outcome

	Univariate Analysis			Multivariate Analysis		

	OR	95% CI	*P*	OR	95% CI	*P*
Age	1.040	1.004-1.077	0.030	--		
Sex	0.249	0.121-0.510	< 0.001	0.042	0.004-0.504	0.012
Systolic blood pressure	1.014	0.999-1.030	0.075	--		
NIHSS score	1.776	1.503-2.099	< 0.001	2.612	1.615-4.224	< 0. 001
Hypertension	0.409	0.256-1.175	0.100	--		
Atrial fibrillation	6.134	0.796-47.242	0.082	--		
Diabetes mellitus	0.601	0.28-1.292	0.193	--		
Smoking	2.568	0.175-5.610	0.018	--		
Alcohol abuse	9.796	2.272-42.244	0.002	--		
WBC (*10^9^/L)	1.093	0.929-1.287	0.284	-		
Lithic acid (μmol/L)	0.997	0.993-1.001	0.092	--		
Vit D (nmol/L)	0.990	0.977-1.005	0.183	--		
TC (mmol/L)	1.051	0.758-1.458	0.766	--		
TG (mmol/L)	0.851	0.641-1.181	0.344	--		
HDL (mmol/L)	1.088	0.334-3.549	0.888	--		
LDL (mmol/L)	1.221	0.824-1.809	0.320	--		
IgG (mg/mL)	1.083	0.955-1.229	0.214	--		
IgA (mg/mL)	1.391	1.046-1.851	0.023	--		
IgM (mg/mL)	1.164	0.721-1.877	0.534	--		
HbA1c (%)	1.263	1.043-1.530	0.017	--		
Glucose (mmol/L)	1.133	1.010-1.270	0.033	--		
Hs-CRP (mg/L)	1.163	1.066-1.270	0.001	1.561	1.12-2.177	0.009
IL-33(ng/L)	0.952	0.931-0.973	< 0.001	0.932	0.882 -0.986	0.006

OR, Odds ratio; CI, Confidence interval; IQR, Interquartile range; NIHSS, National Institutes of Health Stroke Scale; WBC, Leukocyte; Vit D, Vitamin D; TC, Total cholesterol; TG, Triglycerides; HDL, High-density lipoproteins; LDL, Low-density lipoproteins; IgA, Immunoglobulin A; IgG, Immunoglobulin G; IgM, Immunoglobulin M; Hs-CRP, High-sensitivity C-reactive protein; HbA1c, Glycated hemoglobin; IL-33, Interleukin-33.

Based on the ROC curve, the optimal cutoff value of serum IL-33 levels as an index to prognosis of outcome was projected to be 39.3 ng/L. It produced a sensitivity of 90.4% and a specificity of 61.4%, with an AUC of 0.720 (95% CI, 0.639-0.801; *P*<0.001); serum IL-33 showed a significantly greater discriminatory ability as compared with sex, NIHSS score, and Hs-CRP (Table 6).

## DISCUSSION

In our study, we find that the serum IL-33 level is significantly increased during a first-ever episode after AIS. Consistently, Liu J *et al* [20] also showed that serum IL-33 was increased after stroke. Hence, the level of IL-33 in the serum can be used as a diagnostic biomarker for AIS. In addition, IL-33 was seen to play a key role in the recovery of stroke because we found that the expression of serum IL-33 was significantly higher in patients with favorable outcome. This is the first time to examine the relationship between serum IL-33 and stroke outcome. We also confirmed that the level of serum IL-33 was an independent prognostic marker of functional outcome in AIS patients 3 months after stroke onset. Furthermore, the level of serum IL-33 was associated with the severity of stroke. We found that serum IL-33 levels increased significantly, along with descreased NIHSS score and infarct volume. Studies reported that the increased production of anti-inflammatory cytokines after stroke was correlated with a lower NIHSS score and smaller infarct size in animal models and in clinical trials [11, 33]. Thus, we speculate that IL-33 is a protective factor for AIS.

Inflammatory and immune responses play important roles after AIS [8, 34]. In our study, WBC, lithic acid, TC, TG, HDL, LDL, IgG, and IgM showed no significant difference between patients with favorable and unfavorable outcomes. HbA1c, IgA, and glucose were associated with functional outcome, but they were not predictors for outcome of AIS. Only hs-CRP and IL-33 were independent prognostic markers of outcome in AIS patients. Hs-CRP reflected the level of inflammatory response after stroke. Recent reports showed that the level of CRP could predict patient outcomes in cardiovascular disease [35, 36]. We found that Hs-CRP was significantly higher in AIS patients and patients with unfavorable outcome. However, the relationship between serum IL-33 levels and outcomes persisted on additional adjustment for Hs-CRP, and there was weak negative correlation between serum IL-33 and Hs-CRP. Taken together, the data suggest that Hs-CRP and serum IL-33 contribute to the inflammatory response as inflammatory biomarkers, but they are also involved in different signaling pathways. Therefore, these underlying mechanisms need to be further determined.

Whether serum IL-33 in AIS patients is involved in the recovery process or is as an index of anti-inflammation is important. The inflammatory responses after stroke have dual functions. Inflammation may lead to injury after stroke, but may also contribute to recovery processes [9]. A number of studies suggest that anti-inflammatory factors could down-regulate the “bad” actions of pro-inflammatory factors [11,33,37]. Hence, the balance between anti-inflammatory and pro-inflammatory factors is critical in determining the clinical outcome of AIS. The expression of anti-inflammatory players could reduce ischemic tissue damage [33,38, 39].

Our study showed that the patients with increased circulating IL-33 in the serum had better clinical outcome at 3 months, suggesting that IL-33 may be an anti-inflammatory factor and can predict the prognosis of AIS. Hence, we speculated that IL-33 signaling might play an important pathophysiologic and anti-inflammatory role in the process of AIS. The protective role of IL-33 in AIS may be related to the following pathways: first, T cells and their subsets are involved in inflammation-mediated brain injury. Th1 cells promote inflammation and the following secondary brain damage while Th2 cells promote anti-inflammatory responses that reduces secondary brain injury. Many reports hint that the IL-33/ST2 signaling pathway could tilt the Th1/Th2 balance in favor of Th2 cells [18, 40]. IL-33 may also induce Th2 cells to produce Th2-type cytokines such as IL-10, which is an anti-inflammatory cytokine involved in neuroprotection and regeneration [41]. In addition, the increased expressions of important pro-inflammatory cytokines such as IFN-γ and IL-17 contribute to inflammation and ischemic brain damage after stroke [42]. IL-33 may reduce the production of IFN-γ and IL-17 through inhibiting the actions of Th1 and Th17 cells. The exact mechanisms are still unclear and will need further study.

**Table 6:** Prediction of clinic outcome

Prediction	AUC	95% CI	*P*
Sex	0.334	0.239-0.430	0.001
NIHSS score	0.107	0.056-0.157	< 0.001
Hs-CRP	0.290	0.197-0.382	< 0.001
IL-33	0.720	0.639-0.801	< 0.001

AUC, Area under the curve; CI, Confidence interval; NIHSS, National Institutes of Health Stroke Scale; Hs-CRP, High-sensitivity C-reactive protein; IL-33, Interleukin-33.

Current knowledge suggests that treatment with anti-inflammatory agents minimizes the infarction size and increases therapeutic window against ischemic stroke [43, 44]. Therefore, anti-inflammatory therapy may help functional recovery and reparation after ischemic brain injury. A recent study found that IL-33 could play a protective role after ischemic stroke in animals; IL-33 treatment via the intracerebroventricular route decreased infarction size [24]. We found that lower serum IL-33 was associated with an increased stroke severity in AIS patients. Therefore, IL-33 may be a potential therapeutic target for novel therapeutics for the prognosis of AIS.

There are some limitations in this study. Firstly, we only measured circulating IL-33 levels once; therefore, do not know the dynamic change of serum IL-33 at different stage of AIS. Secondly, we detected IL-33 in serum, but not in the cerebral spinal fluid (CSF). Thus, whether the alternation of circulating IL-33 levels is the same as that of the CNS is still unknown.

In conclusion, our study found that increased serum IL-33 level might be used as a novel diagnostic marker and also a useful tool to predict prognosis in patients with acute ischemic stroke at admission.
